# COP1 enhances ubiquitin-mediated degradation of p27^Kip1^ to promote cancer cell growth

**DOI:** 10.18632/oncotarget.3821

**Published:** 2015-04-14

**Authors:** Hyun Ho Choi, Liem Phan, Ping-Chieh Chou, Chun-Hui Su, Sai-Ching J. Yeung, Jiun-Sheng Chen, Mong-Hong Lee

**Affiliations:** ^1^ Department of Molecular and Cellular Oncology, The University of Texas MD Anderson Cancer Center, Houston, TX, USA; ^2^ Institute of Biosciences and Technology, Texas A&M University Health Science Center, Houston, TX, USA; ^3^ Program in Cancer Biology, The University of Texas Graduate School of Biomedical Sciences at Houston, Houston, TX, USA; ^4^ Program in Genes and Development, The University of Texas Graduate School of Biomedical Sciences at Houston, Houston, TX, USA; ^5^ Department of Cancer Emergency Medicine, The University of Texas MD Anderson Cancer Center, Houston, TX, USA

**Keywords:** p27, COP1, ubiquitination, cell cycle

## Abstract

p27 is a critical CDK inhibitor involved in cell cycle regulation, and its stability is critical for cell proliferation. Constitutive photomorphogenic 1 (COP1) is a RING-containing E3 ubiquitin ligase involved in regulating important target proteins for cell growth, but its biological activity in cell cycle progression is not well characterized. Here, we report that p27^Kip1^ levels are accumulated in G1 phase, with concurrent reduction of COP1 levels. Mechanistic studies show that COP1 directly interacts with p27 through a VP motif on p27 and functions as an E3 ligase of p27 to accelerate the ubiquitin-mediated degradation of p27. Also, COP1-p27 axis deregulation is involved in tumorigenesis. These findings define a new mechanism for posttranslational regulation of p27 and provide insight into the characteristics of COP1-overexpressing cancers.

## INTRODUCTION

Constitutive photomorphogenic 1 (COP1) acts as an E3 ubiquitin ligase and has a pivotal role in light signaling in plants, but its role in mammals is much more complex. Plant COP1 can repress light signaling by targeting transcription factors, such as HY5, HYH, for ubiquitination and degradation [1]. Mammalian COP1 is involved in various cellular functions, such as proliferation and survival, by facilitating the degradation of physiological substrates through ubiquitin-mediated protein degradation [2, 3]. COP1 itself is self-ubiquitinated, and this process is under the regulation of COP9 signalosome subunit 6 (CSN6) [4], a protein involved in MDM2-p53 signaling [5] and Cullin neddylation [6]. So far, mammalian E3 ubiquitin ligase COP1 substrates include c-Jun [7], ETV1 [8], p53 [9], (C/EBPalpha) [10-12], acetyl-CoA carboxylase [13], TORC2 [14], MTA1 [15], 14-3-3 σ [4, 16, 17] and FOXO1 [18], suggesting that COP1 is engaged in many biological activities [3]. COP1 is a RING finger protein with coiled-coil and WD 40 domains that associate with target proteins and participates in various biological functions [1]. However, its many substrates remain to be characterized. COP1 regulates p53 and 14-3-3 σ stability to regulate cell growth, but its role in cell cycle regulation is not fully characterized.

The tumor suppressor p27 governing CDK activity is critical for regulating the cell cycle transition from the G0/G1 to the S phase [19] and is a downstream target of oncogenic signals [20-26]. Levels of p27 are tightly regulated to control cell cycle progression [19, 27], and p27 expression levels are protected by the activity of many tumor suppressors [24, 28]. p27 levels are high during the G0 /G1 phase and get reduced when the cells enter into the S phase. p27 levels are mainly regulated through polyubiquitination [29], and they are downregulated in many types of cancer. However, this deregulation in cancers is not completely understood.

Here, we show that p27 levels are reduced in response to COP1 accumulation during cell cycle progression. We show that COP1 directly associates with p27 and functions as an E3 ligase of p27 to accelerate the ubiquitin-mediated degradation of p27 via its RING domain. Also, COP1-mediated p27 downregulation does not require the participation of known regulators of p27 stability: KPC1, Jab1, PirH2 or Skp2. Importantly, the deregulation of COP1-p27 axis manifests in cell proliferation, transformation, and tumorigenicity.

## RESULTS

### COP1 directly interacts with p27 during cell cycle progression

**Figure 1:**
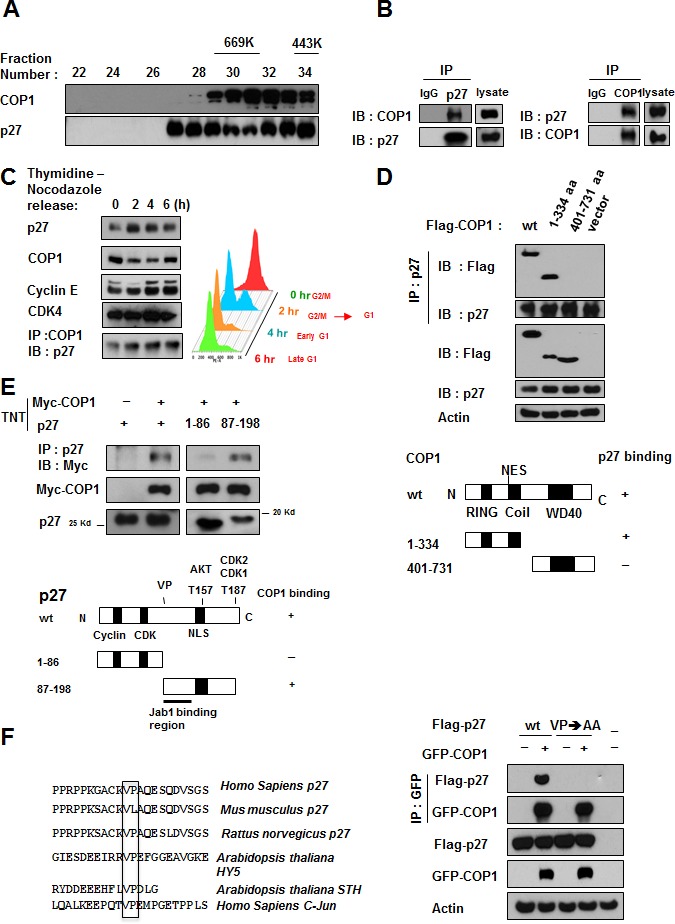
COP1 directly interacts with p27 (**A**) COP1 and p27 coeluted as part of the high molecular weight complex. Gel filtration and elution profiles analysis of COP1 and p27. The distributions of these proteins were analyzed by gel filtration chromatography (Superose 6). Immunoblots of the fractions for indicated proteins are shown in U2OS cells. Molecular size of eluted fraction is indicated above. (**B**) Endogenous interaction of COP1 and p27 was observed. Lysates of U2OS cells were prepared and equal amounts of cell lysates were analyzed by immunoprecipitation (IP) with either control mouse IgG or p27 and analyzed by immunoblotting (IB) with anti-COP1. Lysates were also analyzed by IP with the indicated antibodies and IB with anti-p27. (**C**) p27 and COP1 interacted during the cell cycle. U2OS cells were synchronized to the G2/M phase using treatment with thymidine-nocodazole. Lysates of synchronized cells were analyzed by IB with the indicated antibodies. COP1-p27 interaction at various phases of the cell cycle was detected by IP with the COP1 antibody followed by IB with anti-p27 (left). Cell samples at labeled time points after release of nocodazole were stained with propidium iodide and analyzed by FACS for DNA content. DNA content histograms are shown for the time points indicated (right). (**D**) p27 bound to the N-terminus of COP1 but not to the C-terminus. Wild-type (wt; aa 1-731), N-terminal (aa 1-334), or C-terminal (aa 401-731) Flag-COP1 was transfected into HeLa cells. Cells were treated with MG132, and cell lysates were analyzed by IP with anti-p27 and IB with anti-Flag. (**E**) COP1 bound to the C-terminus of p27. Myc-COP1 and PET-p27 were transcribed and translated *in vitro* (TNT). COP1 and p27 proteins were incubated overnight and analyzed by IP with anti-p27 followed by IB with anti-Myc. (**F**) The interaction of COP1 and p27 was mediated by the conserved VP sequence on p27. p27 has the VP motif for COP1 binding. Consensus COP1 binding motif, highlighted in sequences of p27 in human and other species' DNA for comparison. HY5, STH, and c-Jun proteins are known COP1 binding proteins with VP motif. 293T cells were transfected with the indicated plasmids and treated with MG132. Cell lysates were analyzed by IP with anti-GFP and IB with anti-Flag.

COP1 and p27 coeluted as part of the high molecular weight complex in gel filtration studies (Figure 1A). On the basis of these findings, we hypothesized that COP1 and p27 have an interactive or regulatory relationship. Further, co-immunoprecipitation experiments showed endogenous interaction of the two proteins in cells (Figure 1B). To determine whether the interaction was regulated by the cell cycle, we collected cell lysates from synchronized cells at various time points after release from the thymidine-nocodazole block [30] (Figure 1C). COP1 protein was detected in every cell cycle phase, but levels were reduced when cells were cycling from G2/M to G1 (Figure 1C). In addition, p27 accumulated following the reduction of COP1 at G1. Co-immunoprecipitation of p27 and COP1 demonstrated that they interacted during the cell cycle (Figure 1C). These results suggest that COP1 may have an as-yet uncharacterized function in the cell cycle that involves interaction with p27.

Next, we mapped the structural regions of COP1 required for its interaction with p27. Results showed that p27 was bound to the N-terminus of COP1 (aa 1-334 containing RING motif) but not to the C-terminus (aa 401-473; Figure 1D). We also mapped the COP1 binding region on p27 *in vitro*. A binding assay using TNT products indicated that the C-terminus of p27 (aa 87-198) was responsible for binding COP1 (Figure 1E).

COP1 preferentially binds to target proteins with the VP motifs [31]. We analyzed the p27 peptide sequence and found a putative COP1 binding motif located in p27 (aa 87-198)(Figure 1F). We predicted that abolishing this binding site at aa 101-102 by mutating the VP motif to alanine (VP→AA) would interfere with p27-COP1 binding. Indeed, co-immunoprecipitation showed that the p27 (VP→AA) mutant lost its binding affinity for COP1 (Figure 1F). These results indicate that direct interaction between COP1 and p27 may be critical for cell cycle progression.

### COP1 negatively regulates p27 protein stability

**Figure 2:**
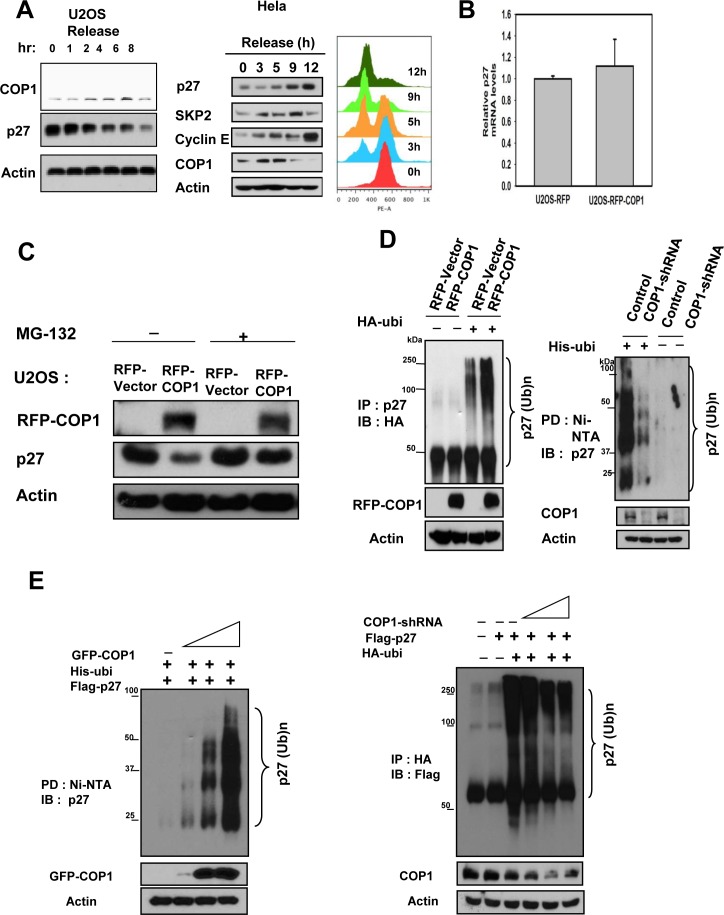
COP1 increases p27 polyubiquitination (**A**) COP1 and p27 levels were inversely correlated in synchronized cells. U2OS cells were synchronized to the G1 phase using treatment with serum starvation, followed by refeeding with the serum. Lysates of synchronized cells after serum refeeding at labeled time points were analyzed by immunoblotting (IB) with the indicated antibodies. HeLa cells were synchronized to the G2/M phase using treatment with thymidine-nocodazole. Lysates of synchronized cells were analyzed by immunoblotting (IB) with the indicated antibodies. Cell samples at labeled time points after release of nocodazole were stained with propidium iodide and analyzed by FACS for DNA content. DNA content histograms are shown for the indicated time points. (**B**) mRNA levels of p27 were not affected by overexpression of COP1. Real-time quantitative PCR analysis of p27 in RFP-COP1–overexpressing U2OS stable transfectants and vector control transfectants showed no obvious difference in p27 mRNA levels.(**C**) MG132 rescued COP1-mediated p27 downregulation. RFP-COP1–overexpressing U2OS stable transfectants and vector control transfectants were treated with or without the proteasome inhibitor MG132 before lysates were collected. Lysates were analyzed by immunoblotting with the indicated antibodies. (**D**-**E**) COP1 regulated endogenous p27 polyubiquitination. Indicated transfected cells were treated with 5 μg/ml MG132 (Sigma) for 6 hours before they were harvested. Cells were lysed in guanidine–HCl containing buffer. The cell lysates then underwent pull down (PD) with nickel beads and analyzed by IB with anti-p27 or was immunoprecipitated (IP) with anti-HA and analyzed by IB with anti-Flag.

We found that COP1 and p27 levels were inversely correlated in synchronized cells after synchronization release (Figure 2A). We showed that mRNA levels of p27 were not affected by COP1 overexpression in a real-time quantitative PCR analysis (Figure 2B) and that COP1-mediated p27 downregulation can be rescued by the proteasome inhibitor MG132 (Figure 2C). These results suggest that COP1 downregulates p27 at the posttranscriptional level.

We then found that overexpression of COP1 increased the endogenous ubiquitination level of p27 (Figure 2D), whereas COP1 knockdown reduced the endogenous ubiquitination level of p27 (Figure 2D). Similar results were obtained for ubiquitination levels of exogenous p27 (Figure 2E). Our data indicate that COP1 is involved in p27 ubiquitination.

### COP1-mediated p27 downregulation requires RING domain and physical binding

**Figure 3:**
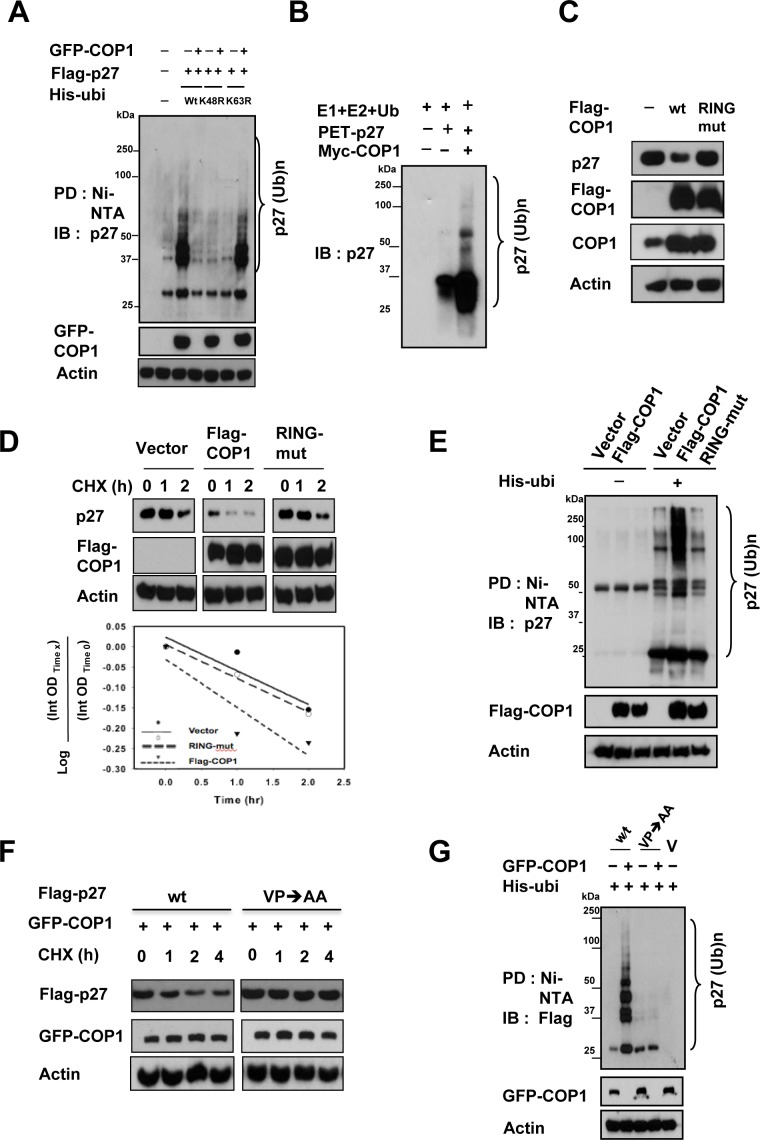
COP1-mediated p27 ubiquitination requires RING domain and physical binding (**A**) COP1-mediated p27 polyubiquitination occurred through the K48 link. The K48R mutant of His-ubi disrupted COP1-mediated p27 polyubiquitination. 293T cells were co-transfected with the indicated plasmids. Cells were treated with 5 μg/ml MG132 (Sigma) for 6 hours before they were harvested. Cells were lysed in guanidine–HCl containing buffer. The cell lysates then underwent pull down (PD) with nickel beads and were analyzed by IB with anti-p27. Equal amounts of cell lysates were analyzed by IB with the indicated antibodies. (**B**) COP1 induced ubiquitination of p27 in an *in vitro* ubiquitination assay. Myc-COP1 and PET-p27 were prepared by *in vitro* transcription and translation using the TNT system. p27 was incubated with or without COP1 in the presence of E1, E2, His-Ubiquitin, and ATP as indicated. The ubiquitinated p27 was detected by IB with anti-p27 antibodies. (**C**) COP1 C136S/C139S (RING mutant) had no impact on p27 levels. HeLa cells were transfected with the indicated expression vectors. Equal amounts of protein from cell lysates were analyzed by IB with anti-p27, or anti-Flag-COP1. (**D**) COP1 C136S/C139S (RING mutant) had no effect on the turnover rate of p27. HeLa cells were transfected with the indicated expression vectors. Forty-eight hours after transfection, the cells were treated with cycloheximide (CHX; 100 μg/ml) for the indicated times. Cell lysates were analyzed by IB with anti-p27, anti-Flag, or anti-Actin. (**E**) Flag-COP1 C136S/C139S (RING mutant) compromised polyubiquitination of p27. HeLa cells were transfected with the indicated expression vectors. Cells were treated with MG132 for 6 hours before they were harvested and lysed in denaturing buffer (6M guanidine-HCl, 0.1M Na_2_HPO_4_/NaH_2_PO_4_, 10mM imidazole). The cell lysates were then incubated with nickel beads for 3 hours, washed, and analyzed by IB with anti-p27. (**F**) The p27 (VP→AA) mutant showed slower turnover in the presence of COP1 than wild-type (wt) p27. 293T cells were co-transfected with GFP-COP1 and either wt Flag-p27 or Flag-p27 (VP→AA). Cells were treated with CHX (100 μg/ml) for the indicated times. Cell lysates were analyzed by IB with the indicated antibodies. (**G**) The p27 (VP→AA) mutant was resistant to COP1-mediated p27 polyubiquitination. 293T cells were co-transfected with the indicated plasmids. Cells were treated with 5 μg/ml MG132 (Sigma) for 6 hours before harvesting. Cells were lysed in guanidine–HCl containing buffer. The cell lysates then underwent PD with nickel beads and were analyzed by IB with anti-Flag. Equal amounts of cell lysates were analyzed by IB with the indicated antibodies.

COP1-mediated p27 polyubiquitination was found to occur through the K48 link (Figure 3A). We also performed an *in vitro* ubiquitination assay to confirm that COP1 E3 ligase is capable of triggering polyubiquitination of p27 (Figure 3B). We then showed that the RING domain of COP1 is responsible for regulating p27 degradation because the COP1 RING mutant (C136S/C139S) was not able to downregulate steady-state expression of p27 (Figure 3C). Congruently, COP1 not only increased the turnover rate of p27 in the presence of the *de novo* protein synthesis inhibitor cycloheximide (Figure 3D), but also facilitated the ubiquitination of p27 (Figure 3E), whereas the COP1 RING mutant (C136S/C139S) failed to increase turnover of p27 and subsequent p27 ubiquitination (Figures 3D & 3E). In line with the binding requirement for COP1-mediated p27 downregulation, the p27 (VP→AA) mutant, which failed to bind COP1, showed slower turnover in the presence of COP1 compared with wt p27 (Figure 3F) and was resistant to COP1-mediated p27 ubiquitination (Figure 3G). In summary, these results demonstrated that COP1-mediated downregulation of p27 occurs through protein-protein interaction on the p27 VP motif and is triggered by COP1 E3 ligase for polyubiquitination through the RING motif.

### COP1-mediated p27 downregulation has impact on cell cycle progression

**Figure 4:**
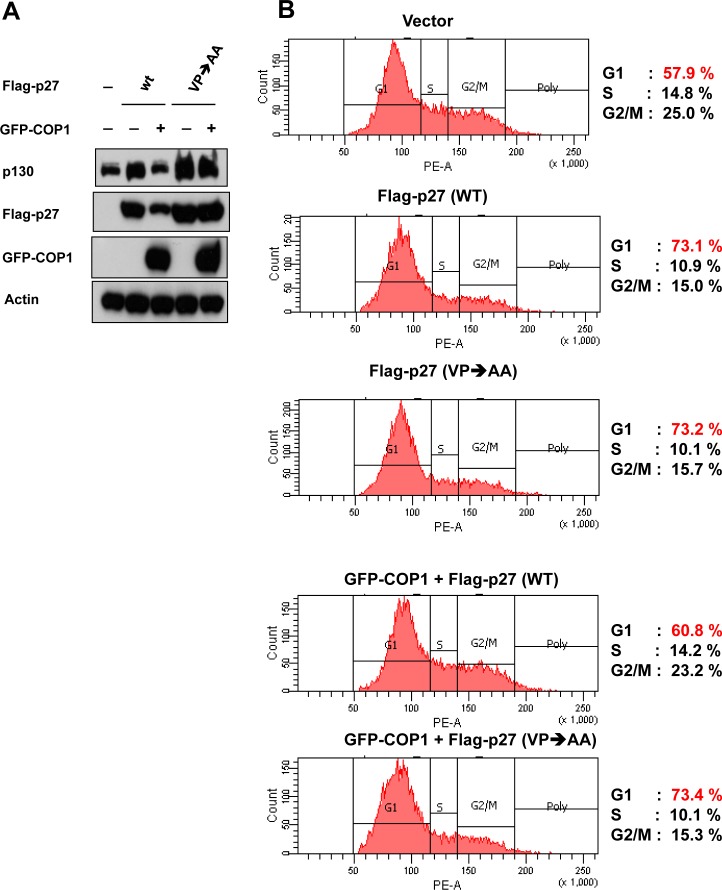
p27 (VP→AA) efficiently mitigates COP1-mediated cell cycle progression (**A**) p27 (VP→AA) was resistant to COP1-meditated degradation. 293T cells were co-transfected with the indicated plasmids. Cell lysates were analyzed by immunoblotting with anti-p130, anti-Flag-p27, anti-GFP-COP1, or Actin antibodies. (**B**) p27 (VP→AA) diminished COP1-mediated cell cycle progression better than wt p27. 293T cells were co-transfected with the indicated plasmids as shown in A. Cells were analyzed for cell-cycle distribution using fluorescence-activated cell sorting.

**Figure 5:**
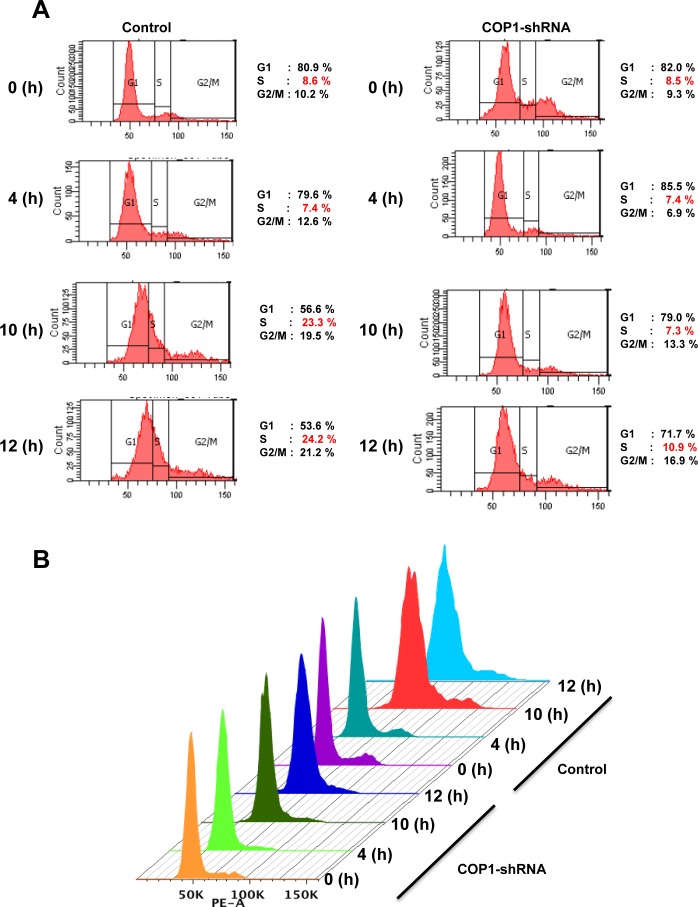
Cell cycle progression is delayed with COP1 deficiency (**A**) COP1 knockdown led to a delay of cell cycle progression. MDA-MB231 cells infected with either lentiviral COP1 shRNA or control shRNA were synchronized in the G0/G1 phase after 72 hours of serum starvation before they were released into fresh medium containing serum. Cells were collected at the indicated time points. (**B**) DNA histograms from A are shown.

We further showed that the steady–state expression of p27 (VP→AA) mutant is not affected by the COP1 (Figure 4A). In contrast, COP1 reduced the steady-state expression of wt p27. As expected, p27 (VP→AA) mutant is still capable of causing G1 arrest (Figure 4B). Importantly, the biological significance of the resistance of p27 (VP→AA) to COP1-mediated degradation is that p27 (VP→AA) can diminish COP1-mediated cell cycle progression better than wt p27 (Figure 4B). COP1 knockdown leads to reduction of p27 ubiquitination (Figure 2), suggesting that COP1 knockdown will increase the level of p27. Because p27 causes cell cycle arrest, the biological significance of p27 elevation caused by COP1 knockdown was a delay of cell cycle progression as demonstrated by fluorescence-activated cell sorting (Figure 5). These data indicate that COP1-p27 link is involved in cell cycle regulation.

### COP1-mediated p27 downregulation is independent of other known E3 ligases of p27

**Figure 6:**
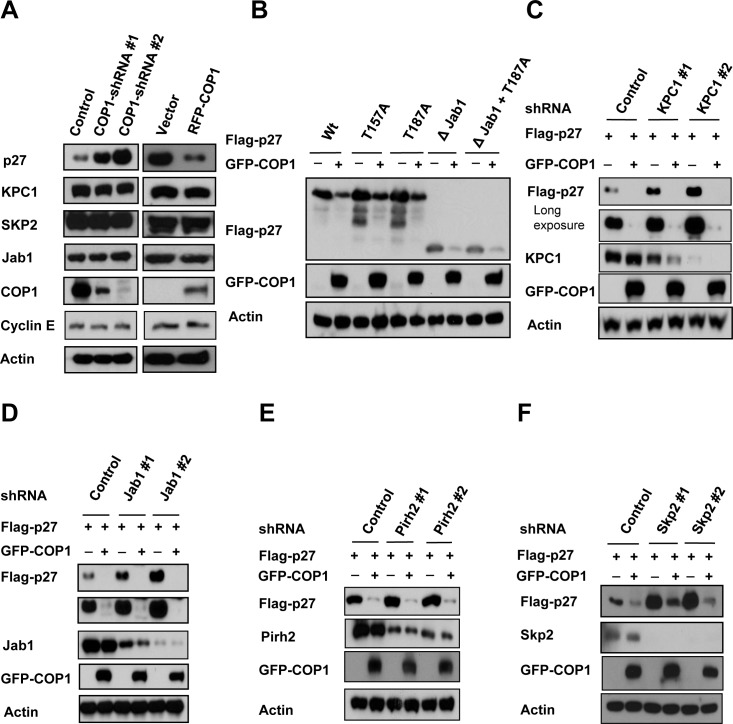
KPC1, Jab1, Pirh2, and SKP2 are not involved in COP1-mediated p27 downregulation (**A**) Protein levels of KPC1, Jab1, Pirh2, and SKP2 were not changed when endogenous COP1 expression was inhibited with shRNA (left). Lysates of MDA-MB231 cells infected with either COP1-shRNA or control shRNA were analyzed by IB with the indicated antibodies. Lysates of COP1-overexpressing U2OS stable transfectants and vector control transfectants were analyzed by IB with the indicated antibodies (right). (**B**) 293T cells were co-transfected with the indicated plasmids. Lysates of cells transfected with indicated plasmids were analyzed by immunoblotting with the indicated antibodies. (**C**)-(**F**) 293T cells were co-transfected with the indicated plasmids. Lysates of cells infected with indicated shRNAs were analyzed by immunoblotting with the indicated antibodies.

We showed that COP1 negatively regulated p27 expression, and we found that p27 levels were elevated when cells were treated with COP1-shRNA virus to perform COP1 knockdown (Figure 6A). Importantly, we also noted that expression levels of several known regulators involved in p27 degradation, including KPC1 [32], SKP2 [29], PirH2 [33], and Jab1 [34, 35], were not affected by the COP1 deficiency or overexpression in this process (Figure 6A). We noted that COP1-mediated p27 downregulation did not involve Akt-mediated phosphorylation (phosphorylation at T157), Cdk2-mediated phosphorylation (phosphorylation at T187 required for Skp2 binding) or Jab1 interaction (required Jab1-binding site on p27) (Figure 6B), as wt p27 and all these different mutants of p27 were downregulated by COP1. In assays using knockdown of KPC1, Jab1, PirH2, or SKP2, we found that COP1-mediated p27 downregulation did not require the participation of KPC1, Jab1, PirH2, or SKP2 (Figures 6C-6F). Together, these data indicate that COP1-mediated p27 downregulation is independent of these regulators.

### COP1-p27 axis regulates cell proliferation and tumor growth

**Figure 7:**
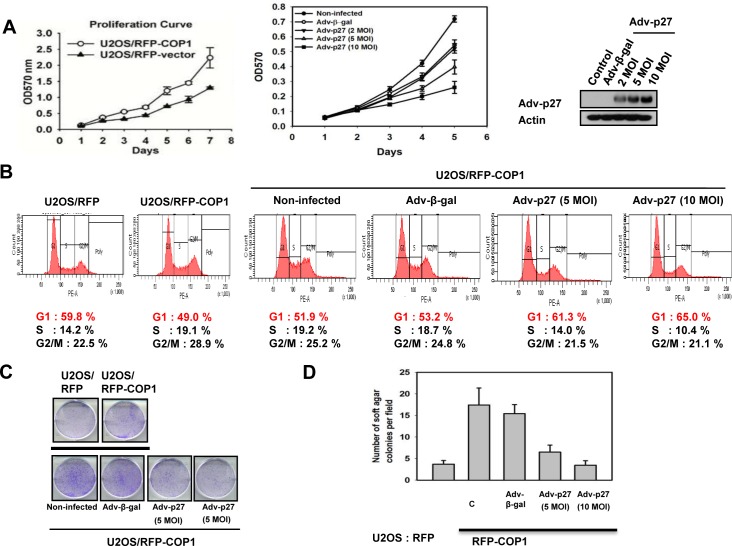
COP1-p27 axis in cell proliferation and transformation (**A**) p27 inhibited COP1-mediated cell proliferation. RFP-COP1 overexpressing U2OS stable transfectants and vector control transfectants were estimated by MTT assay. Also, RFP-COP1 overexpressing cells were infected with Ad-β-gal or Ad-p27 and assayed by MTT assay. Error bars represent 95% confidence intervals. Expression of p27 was shown by Western blot. MOI (multiplicity of infection). (**B**) p27 increased G1 population in RFP-COP1 overexpressing cells. Cells were infected with Ad-β-gal or Ad-p27. Cell cycle distribution was determined using propidium iodide (PI). Cells were stained with PI and analyzed with a FACScalibur flow cytometer. (**C**) –(**D**) p27 replenishment antagonized COP1-mediated foci and soft agar colony formation. Indicated cells were infected with Ad-β-gal or Ad-p27 and analyzed for foci formation or soft agar colony formation. Error bars represent 95% confidence intervals.

**Figure 8:**
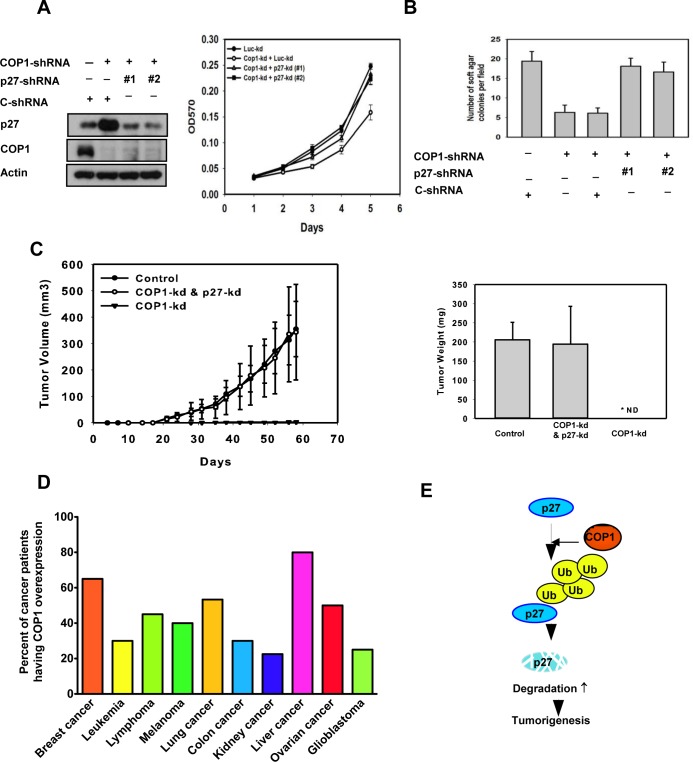
COP1-p27 axis in tumorigenesis (**A**-**B**) COP1-knockdown reduced cell growth and soft agar colony formation. Indicated MDA-MB-231 cells with COP1 or p27 knockdown were analyzed for cell growth by MTT assay or assessed for transformation by soft agar colony formation. Error bars represent 95% confidence intervals. (**C**) COP1-knockdown suppressed tumorigenesis. COP1-knockdown MDA-MB-231 cells were further knocked down with shp27. Cells were harvested and s.c. injected into the flank of nude mice. Tumor volumes were monitored. Tumor growth curves are shown; error bars represent 95% confidence intervals. Tumor weights from each group were measured. Error bars represent 95% confidence intervals. (**D**) COP1 is frequently overexpressed in many common types of cancer. Human cancer patient datasets were obtained from Oncomine and Gene Expression Omnibus. Data were analyzed using Oncomine analysis tools and Nexus Expression 3.0. Patients with more than a 60% increase in COP1 mRNA levels compared with normal tissues were counted as “COP1 overexpression.” The number of patients analyzed: breast cancer (910), leukemia (127), lymphoma (94), melanoma (83), lung cancer (260), colon cancer (237), kidney cancer (114), liver cancer (197), ovarian cancer (50), and glioblastoma (84). (**E**) A model of the relationship between COP1 regulation, p27 stability, and tumor growth is shown.

We further confirmed that COP1-p27 axis could affect cell proliferation. COP1-expression facilitates cell growth (Figure 7A). Since COP1 can mediate p27 inhibition, we sought to examine the growth effect of replenishing p27 in terms of cell proliferation, foci formation, and anchorage-independence in COP1-overexpressing cells. We found that p27 replenishment (through Adenoviral delivery) antagonized COP1-mediated cell proliferation, foci formation, and anchorage-independent growth (Figures 7A-7D). On the other hand, the COP1 knockdown cells have elevated p27 and thus a slower rate of cell proliferation, colony formation, and tumorigenicity (Figures 8A-8C) compared with control cells. Additional p27 knockdown in COP1 knockdown cells reverses COP1 shRNA-mediated reduction of cell proliferation, colony formation, and tumorigenicity (Figures 8A-8C). Significantly, COP1 is overexpressed in many types of cancer (Figure 8D), suggesting that tumors with COP1 overexpression may have growth advantage as shown in our cell and mouse model studies.

## DISCUSSION

COP1 can suppress p53 activity [9] and thus acts as an oncoprotein, but COP1 knockout mouse model data suggest that COP1 may also function as a tumor suppressor via antagonizing oncogenic activity of c-Jun and ETV1 [2, 7, 8] in some tissues; therefore, its role in cancer requires further studies. Importantly, its many substrates remain to be characterized. Further, its role in cell cycle regulation is not clear. A better understanding of the COP1 regulatory complexity is critical for managing cancer therapy. In this study, we found that COP1 could reduce the steady-state expression of p27, a critical CDK inhibitor involved in cell cycle regulation, suggesting that a new layer of regulation on p27 exists in cell cycle progression. The fact that COP1 functions as an E3 ligase of p53 as well as p27, and causes their degradation, suggests an oncogenic role. The COP1 KO mouse model presents a surprising discovery about its tumor suppressor role. Our human cancer sample studies indicate that COP1 is highly elevated in many types of cancer, which is in conflict with tumor suppressive role, suggesting that COP1 knockout mouse cancer studies may have some discrepancies that currently cannot be explained.

The fact that COP1 participates in p27 ubiquitination/degradation is unprecedented as the ubiquitin ligase component F-box protein Skp2 is the major regulator for polyubiquitination of p27 and mediates its degradation [29, 36-39]. However, in the absence of Skp2, p27 is still degraded, suggesting that other E3 ubiquitin ligases may regulate p27 turnover [40]. PirH2, a RING containing protein [41], is the recently identified E3 ligases for p27 [40]. Nonetheless, we still characterized that COP1 is an E3 ligase regulating p27 without the involvement of PirH2, Jab1, KPC1 or Skp2. Further, we showed that COP1 enhances p27 ubiquitination, and that p27 (VP→AA) construct is resistant to COP1-mediated ubiquitination and subsequent degradation, providing important insight into the structure/functional relationship between COP1 and p27.

In our cancer model study, COP1 knockdown inhibited tumor growth in xenograft breast cancer model. Further p27 knockdown in these COP1 knockdown cells reverses COP1 shRNA-mediated reduction of tumorigenicity. Thus, these studies recapitulated the relationship between COP1 and p27 *in vivo*. We also found that *COP1* overexpression is quite common in different types of cancer. p27 downregulation is frequently observed in human cancer samples [26]. Our findings demonstrate that COP1 overexpression can at least partially account for p27 downregulation in many cancers. Together, our results demonstrated a link between COP1 regulation, p27 stability, and cell cycle growth (Figure 8E). The role of COP1 in attenuating p27 offers a new bridge for knowledge gap regarding the role of COP1 in cell cycle and tumorigenicity. Further identifying the structure and functional relationship between COP1 and p27 can provide the basis for developing inhibitors that will block COP1-mediated p27 degradation and functions for rational cancer therapy.

## MATERIALS AND METHODS

### Cell culture and reagents

Human 293T, HeLa, and MDA-MB231 cells were cultured in Dulbecco's modified Eagle medium/F12 medium supplemented with 10% fetal bovine serum, 100 units/ml penicillin G, 100 μg/ml streptomycin, and 0.25 μg/ml amphotericin. U2OS cells were maintained as previously described [42, 43]. For transient transfection, cells were transfected with DNA using either Lipofectamine 2000 (Invitrogen) or FuGENE HD (Roche) reagents according to protocols of the manufacturers. Antibodies to the following epitopes and proteins were purchased from the indicated vendors: Jab1 (GeneTex), Lamin B1 (Abcam), HA (12CA5, Roche), Pirh2 (Bethyl Laboratories), ubiquitin (Zymed Laboratories), p27 (BD Transduction Laboratories and Santa Cruz Biotechnology), and COP1 (Bethyl Laboratories and Santa Cruz Biotechnology). Flag (M2 monoclonal antibody), Tubulin, and Actin were purchased from Sigma. GFP, KPC1, SKP2, Myc (mouse monoclonal 9E10), Cyclin E, and CDK4 were purchased from Santa Cruz Biotechnology.

### Plasmids

pCMV5-Flag-COP1 was kindly provided by E. Bianchi. pcDNA6-Myc-COP1 was constructed by our lab. The COP1 C136S/CS39S mutant was generated using PCR-directed mutagenesis (Stratagene) and verified by DNA sequencing. Wild-type (wt) GFP-COP1, GFP-COP1 NES mutant, and GFP-COP1 S387A were constructed by PCR cloning. The pET15b plasmids expressing Flag-tagged COP1 (aa1-334) and COP1 (aa 401-731) were generated using PCR. pET-p27 wt 1-86, 87-198, Flag-p27 wt, mutants (T157A, T187A, ΔJab1, ΔJab1+T187A) [36], and RFP-p27 Flag-p27 (VP→AA) mutant [44] were generated using PCR-directed mutagenesis.

### Immunoprecipitation and immunoblotting

Total cell lysates were solubilized in lysis buffer (50mM Tris pH 7.5, 150mM NaCl, 1mM EDTA, 0.5% Nonidet P-40, 0.5% Triton X-100, 1mM phenylmethylsulfonyl fluoride, 1mM sodium fluoride, 5mM sodium orthovanadate, and 1 μg/ml each of aprotinin, leupeptin, and pepstatin) and processed as previously described [45, 46]. Lysates were immunoprecipitated with indicated antibodies. Proteins were resolved by SDS-PAGE gels and proteins were transferred to polyvinylidene difluoride membranes (Millipore). The membranes were blocked with 5% nonfat milk for 1 hour at room temperature prior to incubation with indicated primary antibodies. Subsequently, membranes were washed and incubated for 1 hour at room temperature with peroxidase-conjugated secondary antibodies (Thermo Scientific). Following several washes, chemiluminescent images of immunodetected bands on the membranes were recorded on X-ray films using the enhanced chemiluminescence system (Millipore).

### *In vitro* binding assay

For the *in vitro* binding assay, both Myc-COP1 and PET-p27 were prepared by *in vitro* transcription and translation using the TNT system as previously described [45]. TNT protein was mixed and immunoprecipitated with anti-p27 followed by immunoblotting with anti-Myc.

### *In vivo* ubiquitination assay

U2OS, and MDA-MB231 cells were used to detect endogenous p27 ubiquitination as previously described [44]. 293T cells were transiently co-transfected with indicated plasmids to detect exogenous p27 ubiquitination. Forty-eight hours later, cells were treated with 5 μg/ml MG132 (Sigma) for 6 hours before being harvested. Cell lysates were immunoprecipitated with anti-p27 and examined for the levels of p27 ubiquitination. In other cases, cells were lysed in denaturing buffer (6M guanidine-HCl, 0.1M Na_2_HPO_4_/NaH_2_PO_4_, 10mM imidazole). Cell lysates were then incubated with nickel beads for 3 hours, washed, and immunoblotted with anti-p27.

### *In vitro* ubiquitination assay

Experiments were performed as previously described [42, 43]. Both Myc-COP1 and PET-p27 were prepared by *in vitro* transcription and translation using the TNT system.

For detection of ubiquitinated p27 *in vitro*, TNT p27 proteins were incubated with various combinations of ubiquitin (200 pmol), E1 (2 pmol), E2-UbcH5a/5b (10 pmol), *in vitro* translated COP1, and ATP (2mM) in a total volume of 50 μl for 1 hour at 37°C. Reaction products were resolved by 10% SDS-polyacrylamide gel and probed with anti-p27. His-Ubiquitin (UW 8610), E1 (UW 9410), and E2 (UW 9050) were purchased from BioMol International.

### Cell lysates fractionated by gel filtration

Cell lysates were fractionated through a Superose 6 column (GEHealthcare) equilibrated with lysis buffer at a flow rate of 0.3 ml/minute as previously described [6]. Fractions of 300 μl were collected.

### Generation of stable transfectants

To generate stable COP1 knockdown cell lines, MDA-MB231 cells were infected with lentiviral shRNA transduction particles (Sigma; NM_001001740 ring finger and WD repeat domain 2 MISSION shRNA lentiviral transduction particles) containing either control shRNA or COP1 shRNA. After infection, cells were selected with 2 μg/ml puromycin for 2 weeks. For generation of COP1 overexpression stable transfectants, U2OS cells were transfected with indicated plasmids for the generation of stable overexpression transfectants.

### Thymidine-nocodazole block

Experiments were performed as previously described [47].

### FACS cell-cycle analysis

Samples were analyzed using a BD Facscanto II flow cytometer (BD Biosciences) to determine the distribution in different cell-cycle phases (FACS). These experiments were carried out as previously described [47].

### Soft agar colony formation, Foci formation, MTT assay

The experiments were performed as previously described [48].

### Xenograft experiment

Athymic (*nu/nu*) mice were housed in AAALAC-approved barrier facilities. COP1-expressing cells infected with Ad-β-gal (MOI =100) or Ad-p27 (MOI =100) were harvested and injected into the flank of each mouse. Tumor volumes were measured and recorded. At the end of the experiment, the tumors were removed and weighed.
